# Colonization and Diversity of AM Fungi by Morphological Analysis on Medicinal Plants in Southeast China

**DOI:** 10.1155/2015/753842

**Published:** 2015-01-26

**Authors:** Mingyuan Wang, Pan Jiang

**Affiliations:** Department of Horticulture, Huaqiao University, Xiamen 361021, China

## Abstract

The arbuscular mycorrhizal (AM) fungal distributions in the rhizosphere of 20 medicinal plants species in Zhangzhou, southeast China, were studied. The results showed 66 species of 8 genera of AM fungi were identified, of which 38 belonged to *Glomus*, 12 to *Acaulospora*, 9 to *Scutellospora*, 2 to *Gigaspora*, 2 to *Funneliformis*, 1 to *Septoglomus*, 1 to *Rhizophagus*, and 1 to *Archaeospora*. *Glomus* was the dominant genera and *G. melanosporum*, *Acaulospora scrobiculata*, *G. etunicatum*, *Funneliformis mosseae*, and *G. rubiforme* were the prevalent species. The highest colonization (100%) was recorded in *Desmodium pulchellum* (L.) Benth. while the lowest (8.0%) was in *Acorus tatarinowii* Schott. The AM fungi spore density ranged from 270 to 2860 per 100 g soil (average 1005), and the species richness ranged from 3 to 14 (average 9.7) per soil sample. Shannon-Wiener index ranged from 0.52 to 2 (average 1.45). In the present study, the colonization had a highly negative correlation with available *K* and electrical conductivity. Species richness correlated positively with electrical conductivity and organic matter. Shannon-Wiener index had a highly significant negative correlation with pH. This study provides a valuable germplasm and theoretical basis for AM fungal biotechnology on medicinal standardization planting.

## 1. Introduction

Arbuscular mycorrhizal (AM) fungi, the most ubiquitous symbiosis in nature, are a kind of these soil microbes. Reports suggest that estimated 80% of plant species forms mycorrhizas [1]. In general, AM fungi and the host plants are reciprocal symbionts. The symbiosis improves plants the nutrient uptake and provides protection from pathogens, while the AM fungi receive carbohydrates [2–4].

All over the world, 80% of the rural population in developing countries utilizes locally medicinal plants for primary healthcare. And in China, the use of different parts of medicinal plants to cure specific illness has been popular from ancient time. In Zhangzhou, southeast China, the typical humid subtropical monsoon climate contributes to the growth of more than 700 kinds of lush medicinal plants and creates unique ecological conditions for species diversity and distribution of AM fungi.

The distribution of AM fungi associated with medicinal plants has been reported. In a survey on AM association with three different endangered species of* Leptadenia reticulata*,* Mitragyna parvifolia*, and* Withania coagulans*, high diversity of AMF was observed, and* Glomus constrictum*,* Glomus fasciculatum*,* Glomus geosporum*,* Glomus intraradices*,* Glomus mosseae,* and* Glomus rubiforme* were the most dominant species [5]. Similarly, 34 AM fungal species were identified from 36 medicinal plant species [6]. Approximately 15 fungal species from 10 genera were isolated from the collected soils in medicinal plant species, lemon balm (*Melissa officinalis* L.), sage (*Salvia officinalis* L.), and lavender (*Lavandula angustifolia* Mill.) [7]. About 50 species of medicinal plants from 19 families have been studied in the association with AM fungi [8].

However, not enough has been focused on the mycorrhizal association with medicinal plants. Generally, AM fungi species in different ecosystems are affected by edaphic factors, so it is necessary to investigate the spatial distribution and colonization of AM fungi related to the medicinal plants [9–13]. Hence, the present study is attempted to investigate the diversity of AM fungi associated with medicinal plant species in Zhangzhou, southeast China.

## 2. Materials and Methods

### 2.1. Study Sites

The city of Zhangzhou, Fujian province, a subtropical region, is located on 23°08′–25°06′N and 116°53′–118°09′E. The mean annual temperature is 21°C with yearly precipitation of 1000–1700 mm and annual sunshine of 2000–2300 hours. Frost-free periods add up to more than 330 days with cool summer and warm winter. The medicinal plants in this study were collected from Xiaoxi town (24°44′N, 118°17′E), which was cinnamon soil from farmland, and Guoqiang village (24°35′N, 117°56′E), which was cinnamon soil from woodland, in Zhangzhou.

### 2.2. Sample Collection

The plants grew under natural environmental conditions. Six healthy individuals per plant species of medicinal plants (Table 1) were randomly selected for the collection of rhizospheric soil and root samples; 180 soil and root samples were collected from Xiaoxi town and Guoqiang village in October 2011. For each plant, three random soil cores at the depth of 0–30 cm about 1000 g were established by contacting from the 6 duplicate plants. Approximately 20 plants species and 120 soil samples were collected in total. The subsamples were air-dried for 2 weeks and stored in sealed plastic bags at 4°C for the following analysis.

### 2.3. Estimation of AM Colonization

The mixed soil and roots samples of each plant species were packed in polyethylene bags, labeled and brought to the laboratory. The soil samples were air-dried at room temperature. Roots were washed to remove soil particles, preserved with FAA. For colonization measurement, roots were cleared in 10% (w/v) KOH and placed in a water bath (90°C) for 20–30 min. The cooled root samples were then washed with water and stained with 0.5% (w/v) acid fuchsin. Fifty root fragments for each sample (ca. 1 cm long) were mounted on slides in a polyvinyl alcohol solution [14] and examined for the presence of AM structures at 100–400x magnification with an Olympus BX50 microscope for the presence of AM structures. The percentage of root colonization was calculated using the following formula:

(1) root  colonization %=Number  of  arbuscular  mycorrhiza  positive  segmentsTotal  number  of  segments  studied ×100%.

### 2.4. AM Fungus Spore Quantification and Identification

Three aliquots of soil (20 g) were obtained for every plant species. AM fungal spores were extracted from the soil samples by wet sieving and sucrose density gradient centrifugation [15]. Spores were counted under a dissecting microscope, and spore densities (SD) were expressed as the number of spores per 100 g of soil. The isolated spores were mounted in polyvinyl lactoglycerol (PVLG). Morphological identification of spores up to species level was based on spore size, color, thickness of the wall layers, and the subtending hyphae by the identification manual [16] and the website of the International collection of vesicular and AM fungi (http://invam.wvu.edu/).

### 2.5. Soil Analysis

Soil samples were air-dried and sieved through 2 mm grid. Three rhizospheric soil samples (≤2 mm fraction) for each medicinal plant were analyzed for their pH, electrical conductivity (EC), organic matter (OM) content, available N (N), available P (P), and available K (K). Soil pH was measured in soil water suspension 1 : 2 (w/v) by pH meter (PHS-3C, Shanghai Lida Instrument Factory). EC was measured at room temperature in soil suspension (1 : 5 w/v) using conductivity meter (DDS-11C, Shanghai Hong Yi instrument company). OM content was determined by the Walkley-Black acid digestion method. P (extracted with 0.03 M NH_4_F-0.02 M HCl) was measured by molybdenum blue colorimetry, K by an ammonium acetate method using a flame photometer, and N by the alkaline hydrolysis diffusion method [17].

### 2.6. Diversity Studies

Ecological measures of diversity, including spore density (SD), species richness (SR), isolation frequency (IF), Shannon-Wiener index (*H*), and evenness (*J*), were used to describe the structure of AM fungi communities [18, 19]. Diversity studies were carried out from Zhangzhou separately for abundance and diversity of AM fungal species. Spore density was defined as the number of AM fungi spores and sporocarps in 100 g soil; species richness was measured as the number of AM fungi species present in soil sample; isolation frequency (IF) = (number of samples in which the species or genus was observed/total samples) × 100%. Species diversity was assessed by the Shannon-Weiner index as follows: *H* = −∑_*i*=1_^*k*^(*P*_*i*_ln⁡⁡*P*_*i*_); species evenness is calculated by the following formula: *J* = *H*/*H*_max⁡_ where *H*_max⁡_ = −ln⁡*S*, *S* = total number of species in the community (richness). *P*_*i*_ is the relative abundance of each identified species per sampling site and is calculated by the following formula: *P*_*i*_ = *n*_*i*_/*N*, where *n*_*i*_ is the spore numbers of a species and *N* is the total number of identified spore samples. *H*_max⁡_ is the maximal *H* and calculated by the following formula: *H* = ln⁡*S*, where *S* is the total number of identified species per sampling site.

### 2.7. Statistical Analysis

The analysis of Pearson correlation coefficient, variance (ANOVA), and principal component were all carried out with SPSS Bass 18.0 (SPSS Inc., USA). The Pearson correlation coefficient was employed to determine the relationships between AM colonization, SD, SR, IF, *H*, *J*, and soil parameters. Differences in soil parameters, colonization, SD, SR, IF, *H*, and *J* were tested using one-way ANOVA and means were compared by least significant difference at 5% level.

## 3. Results

### 3.1. Soil Parameters

Results of the rhizospheric soil parameters of the 20 medicinal plants harvested at both sites are summarized in Table 2. The soil P ranged from 10.46 mg kg^−1^ to 979.94 mg kg^−1^, the soil K from 28.64 mg kg^−1^ to 184.81 mg kg^−1^, and the soil N from 14.93 mg kg^−1^ to 111.48 mg kg^−1^. The OM ranged from 5.49 g kg^−1^ to 14.44 g kg^−1^. Furthermore, the soil was acidic as the pH ranged from 4.60 to 7.78. EC was 28.15 *μ*s cm^−1^ to 259.75 *μ*s cm^−1^.

### 3.2. AM Colonization, Diversity Index, and Diversity of AM Fungi

Colonization rate, SD, SR, *H*, and *J* of AM fungi in the rhizosphere of 20 medicinal plants species are presented in Table 3. The percentage of root colonization ranged from 8% to 100% with an average of 58.99%. The highest colonization was observed in* Desmodium pulchellum* (L.) Benth. and lowest in* Acorus tatarinowii* Schott. The SD in association with the 20 medicinal plant species ranged from 270 to 2860 spores per 100 g soil, with an average of 1005 spores per 100 g soil. The highest SD was observed in the rhizospheric soil of* Lophatherum gracile* Brongn. and significantly different with in* Leonurus heterophyllus* Sweet f. The highest SR (14) was recorded in* Acorus tatarinowii* Schott., while the lowest (3) appeared in* Leonurus heterophyllus* Sweet f., with a mean of 9.68. The maximum *H* occurred in* Acorus tatarinowii* Schott. (2.00), and the minimum in* Leonurus heterophyllus* Sweet f. (0.52) (average 1.45). The *J* of AM fungi ranged from 0.27 to 0.96 (average 0.66).

The results showed that 66 species of 8 genera of AM fungi were isolated and identified, of which 38 belonged to* Glomus*, 12 to* Acaulospora*, 9 to* Scutellospora*, 2 to* Gigaspora*, 2 to* Funneliformis*, 1 to* Septoglomus*, 1 to* Rhizophagus,* and 1 to* Archaeospora* (Table 4).

Based on IF,* Glomus melanosporum*,* Acaulospora scrobiculata*,* Glomus etunicatum*,* Funneliformis mosseae*, and* Glomus rubiforme* were the prevalent AM fungi in decreasing order (Table 4). Generally, AM fungi with an* IV* greater than 50% were defined as dominant species. So* Glomus melanosporum* was the prevalent AM fungi with the highest IF (100%).* Glomus* was the dominant genus with an IF (100%), followed by* Acaulospora*, IF (95%).

### 3.3. Correlation Analysis

As the soil characteristics may play a key role in the ecological distribution of AM fungi, P, K, N, OM, pH, and EC of the soil samples were investigated. In the present study, the colonization was negatively correlated with AK and EC, but positively correlated with OM. Spore density was positively correlated with OM. The same correlation was found between SR and N, EC and OM. *H* was negatively correlated with pH, whereas *J* was negatively correlated with SD (Table 5).

## 4. Discussion

In the present study, the composition and diversity of the AM fungi composition were described based on morphological species. The results indicated that* Glomus* was the dominant genus, followed by* Acaulospora*.* Acaulospora* and* Glomus* species usually produce more spores than* Gigaspora* and* Scutellospora* species in the same environment [20, 21]. This may be explained by the difference in development.* Acaulospora* and* Glomus* species are thought to require less time to produce spores than* Gigaspora* and* Scutellospora* species. Furthermore, members of the* Gigasporaceae* typically establish an extensive mycelium in soil and produce fewer spores than those of the* Acaulosporaceae* and* Glomaceae* [22, 23].

The results showed a strong symbiotic relationship between 20 medicinal plants and AM fungi, but significant differences were observed in the different plant species. As the studies have shown nonrandom differences in distribution among different AM fungi species and genera in the field, it is also likely that the preferences of different AM fungi for different host plants in our study might be reflected at the species or family level [24, 25].

All 20 medicinal plants were infected by AM fungi, but the degree of colonization and the spore density varied among plant species. This may due to differences in the ability of AM species to sporulate [26]. The host plants used in the trap cultures may also have been an important factor influencing mycorrhizal development, spore formation, and distribution of AM fungi [27]. Many AM species which infect the roots of plants but do not sporulate in the soil may have remained undetected in the present study [28]. Further studies using molecular tools could solve this situation by allowing identification of AM fungi that colonize the roots but remain unsporulating.

AM fungal SD, SR have been positively correlated with OM. OM could enhance spore production [29], extra radical proliferation of hyphae [30], and improve AM colonization [31]. In addition, AM fungal hyphae grew best in soils with a high amount of OM [32]. Soil pH in our study was negatively correlated with AM fungal *H*. Soil pH could affect sporulation, spore germination [33], hyphal growth and root colonization [34], and reproduction and community structure of AM fungi [35]. The range of pH from 5.5 to 6.5 has been found to favour* Glomus* to sporulate more abundantly in acid soils [33].

In the present study, SR was positively correlated with EC. High EC could directly affect the solutes on osmotic potential and delay or prevent all or any of the spore germination phases by dissolved salts in the soil solution. As solution concentrations increased, maximum percent germination and germination rate declined. Effects of salinity on photosynthesis are known to differ between plant species and also between plants at different stages of development [35].

The AM colonization and diversity of medicinal plants in southeast China were investigated in the present study. From the research, we could conclude that the biodiversity of AM fungi was abundant, though* Glomus* was the dominant genus. The degree of colonization and the spore density varied markedly among plant species. Considering the potential application of AM fungi on medicinal plants, it seems that more attention should be paid to the predominant AM fungi during the process of their cultivation, especially mycorrhizal performance (e.g., improving growth, increasing secondary metabolite production).

## Figures and Tables

**Table 1:** The botanic families response to the 20 medicinal plants.

Latin name	Botanic family
*Woodwardia japonica* (L. f.) Sm.	Blechnaceae
*Melastoma candidum* D.Don	Ranunculaceae
*Leonurus heterophyllus* Sweet f.	Labiatae
*Ocimum gratissimum* L. var. *suave* (Willd.) Hook.f.	Labiatae
*Desmodium pulchellum* (L.) Benth.	Papilionaceae
*Lygodium japonicum* (Thunb.) Sw.	Lygodiaceae
*Mentha haplocalyx* Briq.	Labiatae
*Gonostegia hirta* (Blume.) Miq.	Urticaceae
*Gardenia jasminoides* Ellis	Rubiaceae
*Mallotus apelta* (Lour.) Muell.Arg.	Euphorbiaceae
*Antenoron filiforme* Thunb.	Polygonaceae
*Polygonum chinense* L.	Polygonaceae
*Sarcandra glabra* (Thunb.) Nakai	Chloranthaceae
*Pogonatherum crinitum* (Thunb.) Kunth	Agrostidoidaceae
*Selaginella uncinata* (Desv.) Spring.	Selaginellaceae
*Lophatherum gracile* Brongn.	Agrostidoidaceae
*Alpinia officinarum* Hance	Zingiberaceae
*Acorus tatarinowii* Schott.	Araceae
*Ardisia crenata* Sims.	Myrsinaceae
*Citrus medica* L. var. *sarcodactylis* Swingle	Rutaceae

**Table 2:** The rhizospheric soil properties of 20 medicinal plants.

Host plants (20 species)	P/(mg kg^−1^)	K/(mg kg^−1^)	N/(mg kg^−1^)	OM/(g 100 g^−1^)	pH	EC/(*µ*s cm^−1^)
*Woodwardia japonica* (L. f.) Sm.	23.58 ± 2.67	104.78 ± 9.31	22.38 ± 1.49	65.50 ± 4.37	5.36 ± 0.36	54.48 ± 3.63
*Melastoma candidum* D.Don	18.42 ± 2.09	43.88 ± 3.90	14.93 ± 0.99	109.7 ± 7.26	4.84 ± 0.32	82.45 ± 5.50
*Leonurus heterophyllus* Sweet f.	705.79 ± 79.99	95.60 ± 8.50	23.96 ± 1.60	54.9 ± 3.66	7.78 ± 0.52	62.55 ± 4.17
*Ocimum gratissimum* L. var. *suave* (Willd.) Hook.f.	117.65 ± 13.33	64.91 ± 5.76	22.13 ± 1.48	64.7 ± 4.31	5.52 ± 0.37	43.45 ± 2.90
*Desmodium pulchellum *(L.) Benth.	13.98 ± 1.58	57.99 ± 5.15	111.48 ± 7.43	112.3 ± 7.49	6.62 ± 0.44	35.40 ± 2.36
*Lygodium japonicum* (Thunb.) Sw.	22.68 ± 2.57	39.48 ± 3.51	15.56 ± 1.04	59.6 ± 3.97	5.50 ± 0.36	28.15 ± 1.88
*Mentha haplocalyx* Briq.	979.94 ± 111.06	83.86 ± 7.45	29.33 ± 1.96	154.9 ± 10.33	6.29 ± 0.42	72.95 ± 4.87
*Gonostegia hirta* (Blume.) Miq.	16.96 ± 1.92	48.09 ± 4.27	25.22 ± 1.68	103.4 ± 6.89	4.74 ± 0.31	58.75 ± 3.92
*Gardenia jasminoides* Ellis	43.85 ± 4.97	39.32 ± 3.50	36.41 ± 2.43	100.8 ± 6.72	5.41 ± 0.36	41.35 ± 2.76
*Mallotus apelta* (Lour.) Muell.Arg.	34.13 ± 3.86	35.17 ± 3.13	28.51 ± 1.90	80.9 ± 5.39	5.30 ± 0.35	42.00 ± 2.80
*Antenoron filiforme *Thunb.	205.89 ± 23.33	65.41 ± 5.81	29.93 ± 2.00	101.7 ± 6.78	5.43 ± 0.36	52.35 ± 3.49
*Polygonum chinense* L.	50.30 ± 5.70	64.90 ± 5.77	25.05 ± 1.67	100.8 ± 6.71	5.44 ± 0.29	49.10 ± 3.27
*Sarcandra glabra* (Thunb.) Nakai	18.48 ± 2.09	56.29 ± 5.00	22.38 ± 1.49	111.1 ± 7.41	5.02 ± 0.33	66.20 ± 4.41
*Pogonatherum crinitum* (Thunb.) Kunth	10.46 ± 1.19	28.64 ± 2.55	15.51 ± 1.03	87.4 ± 5.83	5.27 ± 0.35	35.70 ± 2.38
*Selaginella uncinata* (Desv.) Spring.	23.29 ± 2.64	71.83 ± 6.38	23.91 ± 1.59	104.5 ± 6.97	5.50 ± 0.37	36.90 ± 2.46
*Lophatherum gracile* Brongn.	18.04 ± 2.04	47.46 ± 4.22	27.53 ± 1.84	108.4 ± 7.23	4.92 ± 0.32	46.35 ± 3.09
*Alpinia officinarum* Hance	74.51 ± 8.44	36.76 ± 3.27	42.02 ± 2.80	132.9 ± 8.86	4.60 ± 0.30	85.15 ± 5.67
*Acorus tatarinowii* Schott.	240.54 ± 27.26	184.81 ± 16.43	39.31 ± 2.62	109.4 ± 7.29	5.45 ± 0.39	259.75 ± 17.32
*Ardisia crenata* Sims.	26.29 ± 2.98	43.22 ± 3.84	47.63 ± 3.18	144.4 ± 9.63	4.66 ± 0.42	66.90 ± 4.89
*Citrus medica* L. var. *sarcodactylis* Swingle	181.99 ± 20.63	70.94 ± 6.31	47.71 ± 3.34	128.7 ± 8.58	5.92 ± 0.51	93.00 ± 8.20

P: available P; K: available K; N: available N; OM: organic matter; EC: electrical conductivity; means of six replicates ± standard deviation.

**Table 3:** The AM colonization and diversity index of 20 species of medicinal plants.

Host plant	Colonization (%)	SD/100 g soil	SR	*H*	*J*
*Woodwardia japonica* (L. f.) Sm.	19 ± 1.73	670 ± 74.44	13 ± 1.41	1.55 ± 0.14	0.60 ± 0.05
*Melastoma candidum* D.Don	27 ± 2.46	1090 ± 121.11	7 ± 0.78	1.36 ± 0.12	0.70 ± 0.06
*Leonurus heterophyllus* Sweet f.	12 ± 1.09	270 ± 30	3 ± 0.23	0.52 ± 0.05	0.48 ± 0.04
*Ocimum gratissimum* L. var. *suave* (Willd.) Hook.f.	65 ± 5.92	680 ± 75.56	10 ± 1.11	1.79 ± 0.16	0.78 ± 0.07
*Desmodium pulchellum *(L.) Benth.	100 ± 9.11	1330 ± 147.78	11 ± 1.23	1.17 ± 0.10	0.49 ± 0.04
*Lygodium japonicum* (Thunb.) Sw.	40 ± 3.64	760 ± 84.87	11 ± 1.09	1.72 ± 0.15	0.72 ± 0.06
*Mentha haplocalyx* Briq.	85 ± 7.74	910 ± 101.12	11 ± 0.99	1.57 ± 0.14	0.65 ± 0.06
*Gonostegia hirta* (Blume.) Miq.	69 ± 6.29	700 ± 77.78	10 ± 1.01	1.24 ± 0.11	0.54 ± 0.04
*Gardenia jasminoides* Ellis	42 ± 3.83	1450 ± 161.78	11 ± 1.22	1.61 ± 0.14	0.67 ± 0.05
*Mallotus apelta* (Lour.) Muell.Arg.	64 ± 5.83	660 ± 73.33	11 ± 1.31	1.84 ± 0.16	0.74 ± 0.06
*Antenoron filiforme* Thunb.	86 ± 7.84	810 ± 90	9 ± 1.00	0.99 ± 0.09	0.47 ± 0.04
*Polygonum chinense* L.	97 ± 8.85	540 ± 60	6 ± 0.68	1.37 ± 0.12	0.79 ± 0.07
*Sarcandra glabra* (Thunb.) Nakai	64 ± 5.84	770 ± 85.66	9 ± 0.89	1.66 ± 0.14	0.76 ± 0.08
*Pogonatherum crinitum* (Thunb.) Kunth	62 ± 5.65	360 ± 40	5 ± 0.55	1.54 ± 0.14	0.96 ± 0.08
*Selaginella uncinata* (Desv.) Spring.	70 ± 6.38	990 ± 110	8 ± 0.85	1.27 ± 0.11	0.61 ± 0.05
*Lophatherum gracile* Brongn.	92 ± 8.38	2860 ± 317.98	8 ± 0.78	1.21 ± 0.10	0.58 ± 0.05
*Alpinia officinarum* Hance	95 ± 8.66	1160 ± 128.32	13 ± 1.54	1.95 ± 0.19	0.76 ± 0.07
*Acorus tatarinowii* Schott.	8 ± 0.73	680 ± 75.67	14 ± 1.56	2.00 ± 0.21	0.76 ± 0.07
*Ardisia crenata* Sims.	60 ± 5.47	980 ± 108.89	12 ± 1.12	1.98 ± 0.18	0.80 ± 0.07
*Citrus medica* L. var. *sarcodactylis* Swingle	21 ± 1.91	2420 ± 268.45	11 ± 1.08	0.64 ± 0.07	0.27 ± 0.02

SD: spore density; SR: spore richness; *H*: Shannon-Weiner index; *J*: evenness; means of six replicates ± standard deviation.

**Table 4:** Isolation frequency (IF) of AM fungi.

Arbuscular mycorrhizal fungi species	IF%
*Septoglomus *	15 ± 1.10
*Septoglomus constrictum* Trappe	15 ± 1.61
*Archaeospora *	10 ± 0.87
*Archaeospora leptoticha* Skenck & Smith	10 ± 0.90
*Funneliformis *	52 ± 4.78
*Funneliformis mosseae* (Nicolson & Gerdemann) Gerdemann & Trappe	50 ± 4.88
*Funneliformis geosporum* (Nicolson & Gerdemann) Walker	45 ± 4.01
*Gigaspora *	15 ± 1.31
*Gigaspora margarita* Becker & Hall	5 ± 0.43
*Gigaspora ramisporophora* Spain, Sieverding & Schenck	10 ± 0.97
*Rhizophagus *	15 ± 1.21
*Rhizophagus fasciculatum fasciculatum* (Thaxt.) Gerd. & Trappe.	15 ± 1.40
*Scutellospora *	35 ± 3.23
*Scutellospora aurigloba *(Hall) Walker & Sanders	5 ± 0.44
*Scutellospora castanea* Walker	5 ± 0.39
*Scutellospora calospora* Nicolson & Gerd.	5 ± 0.46
*Scutellospora erythropa* Koske & Walker	5 ± 0.61
*Scutellospora heterogama* Nicolon & Gerd.	5 ± 0.48
*Scutellospora nigra* (Redhead) Walker & Sanders	5 ± 0.43
*Scutellospora pellucida* Koske & Walker	5 ± 0.45
*Scutellospora persica* Koske & Walker	5 ± 0.53
*Scutellospora rubra* Stürmer & Morton	5 ± 0.51
*Acaulospora *	95 ± 8.23
*Acaulospora bireticulata* Rothwell & Trappe	20 ± 1.78
*Acaulospora cavernata* Blaszkowski	10 ± 0.88
*Acaulospora delicata* Walker, Pfeiffer & Bloss	5 ± 0.40
*Acaulospora demannii* Schenck & Nicolson	15 ± 1.31
*Acaulospora excavata* Walker & Mason	10 ± 0.91
*Acaulospora foveata* Trappe & Janos	10 ± 0.9440
*Acaulospora gedanensis* Blaszkowki	5 ± 0.48
*Acaulospora lacunosa* Morton	10 ± 0.89
*Acaulospora laevis* Gerdemann & Trappe	5 ± 0.45
*Acaulospora mellea* Sieverding & Howeler	5 ± 0.47
*Acaulospora rehmii* Sieverding & Toro	5 ± 0.39
*Acaulospora scrobiculata* Trappe	70 ± 6.67
*Glomus *	100 ± 11.34
*Glomus aggregatum* Schenck & Smith.	15 ± 1.63
*Glomus albidum* Walker & Rhode	40 ± 3.96
*Glomus arenarium* Blaszkowski, Tadych & Madej	10 ± 0.87
*Glomus aureum* Oehl, Wiemken & Sieverding	10 ± 0.94
*Glomus badium* Oehl, Redecker & Sieverding	20 ± 2.12
*Glomus brohultii* Sieverd & Herrera	5 ± 0.48
*Glomus callosum* Sieverding	5 ± 0.48
*Glomus citricola* Tang & zang	5 ± 0.44
*Glomus claroideum* Trappe & Gerdemann	5 ± 0.41
*Glomus clarum* Nicolson & Gerdemann	10 ± 0.98
*Glomus convolutum* Gerd. & Trappe	40 ± 3.86
*Glomus coremiodes* Redecker & Morton	5 ± 0.43
*Glomus coronatum* Giovannetti, Avio & Salutini	5 ± 0.41
*Glomus deserticola* Trappe, Bloss & Menge	10 ± 0.93
*Glomus diaphanum* Morton & Walker	15 ± 1.45
*Glomus dimorphicum* Boyetchko & Tewari	5 ± 0.43
*Glomus dolichosporum* Zhang, Wang & Xing	20 ± 1.74
*Glomus etunicatum* Becker & Gerdemann	50 ± 4.84
*Glomus formosanum* Wu & Chen	5 ± 0.38
*Glomus globiferum* Koske & Walker	10 ± 0.87
*Glomus heterosporum* Smith & Schenck	15 ± 1.44
*Glomus hyderabadensis* Rani, Kunwar, Prasad & Manoharachary	5 ± 0.50
*Glomus intraradices* Schenck & Smith	15 ± 1.32
*Glomus lamellosum* Dalpe, Koske & Tews	15 ± 1.24
*Glomus luteum* Kennedy, Stutz & Morton	5 ± 0.40
*Glomus macrocarpum* (Tul. & Tul.) Berch & Fortin	5 ± 0.42
*Glomus manihotis* Howeler, Sieverding & Schenck	10 ± 1.09
*Glomus melanosporum* Gerdemann & Trappe	100 ± 9.34
*Glomus microaggegatum* Koske, Gemma & Olexia	15 ± 1.22
*Glomus microcarpum* Tul. & Tul.	5 ± 0.45
G*lomus *monosporum Trappe & Gerd	5 ± 0.45
*Glomus multicaule* Gerdemann & Bakshi.	15 ± 1.33
*Glomus reticulatum* Bhattacharjee & Mukerji	15 ± 1.45
*Glomus rubiforme* (Gerd. & Trappe) Almeida & Schenck	50 ± 4.66
G*lomus sinuosum* Almeida & Schenck	5 ± 0.45
*Glomus verruculosum* Blaszkowski & Tadych	10 ± 0.96
*Glomus versiforme* (Karsten) Berch	10 ± 0.83
*Glomus viscosum* Nicolson	5 ± 0.43

Means of six replicates ± standard deviation.

**Table 5:** Correlation analysis between AM fungi and different edaphic factors.

	P	K	N	OM	pH	EC	Colonization	SD	SR	*H*	*J*
P	1.000										
K	0.393^*^	1.000									
N	−0.064	0.056	1.000								
OM	0.160	−0.023	0.391^*^	1.000							
pH	0.631^**^	0.343^*^	0.292	−0.170	1.000						
EC	0.199	0.792^**^	0.070	0.268	−0.027	1.000					
Colonization	−0.113	−0.479^**^	0.283	0.365^*^	−0.208	−0.442^**^	1.000				
SD	−0.164	−0.164	0.306	0.424^**^	−0.120	−0.017	0.14	1.000			
SR	−0.134	0.250	0.361^*^	0.410^**^	−0.227	0.381^*^	−0.042	0.236	1.000		
*H*	−0.265	−0.001	−0.07	0.200	−0.488^**^	0.240	0.115	−0.216	0.585^**^	1.000	
*J*	−0.214	−0.122	−0.27	0.080	−0.289	0.060	0.141	−0.365^*^	0.114	0.795^**^	1.000

P: available P; K: available K; N: available N; OM: organic matter; EC: electrical conductivity; SD: spore density; SR: spore richness; *H*: Shannon-Weiner index; *J*: evenness; ^*^ *P* < 0.05, ^**^ *P* < 0.01.
